# Acetylcholinesterase (*ace-1*^*R*^) target site mutation G119S and resistance to carbamates in *Anopheles gambiae* (*sensu lato*) populations from Mali

**DOI:** 10.1186/s13071-020-04150-x

**Published:** 2020-06-05

**Authors:** Moussa Keïta, Fousseyni Kané, Oumar Thiero, Boissé Traoré, Francis Zeukeng, Ambiélè Bernard Sodio, Sekou Fantamady Traoré, Rousseau Djouaka, Seydou Doumbia, Nafomon Sogoba

**Affiliations:** 1 Malaria Research and Training Center, International Center for Excellence in Research, Faculty of Medicine and Odonto Stomatology, University of Sciences, Techniques and Technologies of Bamako (USTTB), Bamako, Mali; 2 The AgroEcohealth Platform, International Institute of Tropical Agriculture (IITA-Benin), 08 Tripostal, P.O. Box 0932, Cotonou, Benin; 3 Faculty of Science and Technique, University of Sciences, Techniques and Technologies of Bamako (USTTB), Bamako, Mali

**Keywords:** Malaria, Mali, *Anopheles gambiae* (*s.l.*), Vector control, Insecticide resistance, Resistance mechanism, *ace 1*

## Abstract

**Background:**

The long-lasting insecticidal nets (LLINs) and indoor residual spraying of insecticide (IRS) are major malaria vector control strategies in Mali. The success of control strategies depends on a better understanding of the status of malaria vectors with respect to the insecticides used. In this study we evaluate the level of resistance of *Anopheles gambiae* (*sensu lato*) to bendiocarb and the molecular mechanism that underlies it.

**Methods:**

Larvae of *An. gambiae* (*s.l.*) were collected from breeding habitats encountered in the three study sites and bioassayed with bendiocarb. The *ace-1* target site substitution G119S was genotyped using a TaqMan assay.

**Results:**

The three species of the *An. gambiae* complex in Mali, i.e. *An. arabiensis*, *An*. *coluzzii* and *An. gambiae* (*s.s.*) were found in sympatry in the three surveyed localities with different frequencies. We observed a resistance and suspicious resistance of the three species to bendiocarb with a mortality rate ranging from 37% to 86%. The allelic frequency of the G119S mutation was higher in *An. gambiae* (*s.s.*) compared to the other two species; 42.86%, 25.61% and 16.67% respectively in Dangassa, Koula, and Karadié. The allelic frequency of G119S in *An. coluzzii* ranged from 4.5% to 8.33% and from 1.43% to 21.15% for *An. arabiensis.* After exposure to bendiocarb, the G119S mutation was found only in survivors. The survival of *Anopheles gambiae* (*s.l*) populations from the three surveyed localities was associated with the presence of the mutation.

**Conclusions:**

The study highlights the implication of G119S mutation in bendiocarb resistance in *An. gambiae* (*s.s.*), *An. arabiensis* and *An. coluzzii* populations from the three surveyed localities.
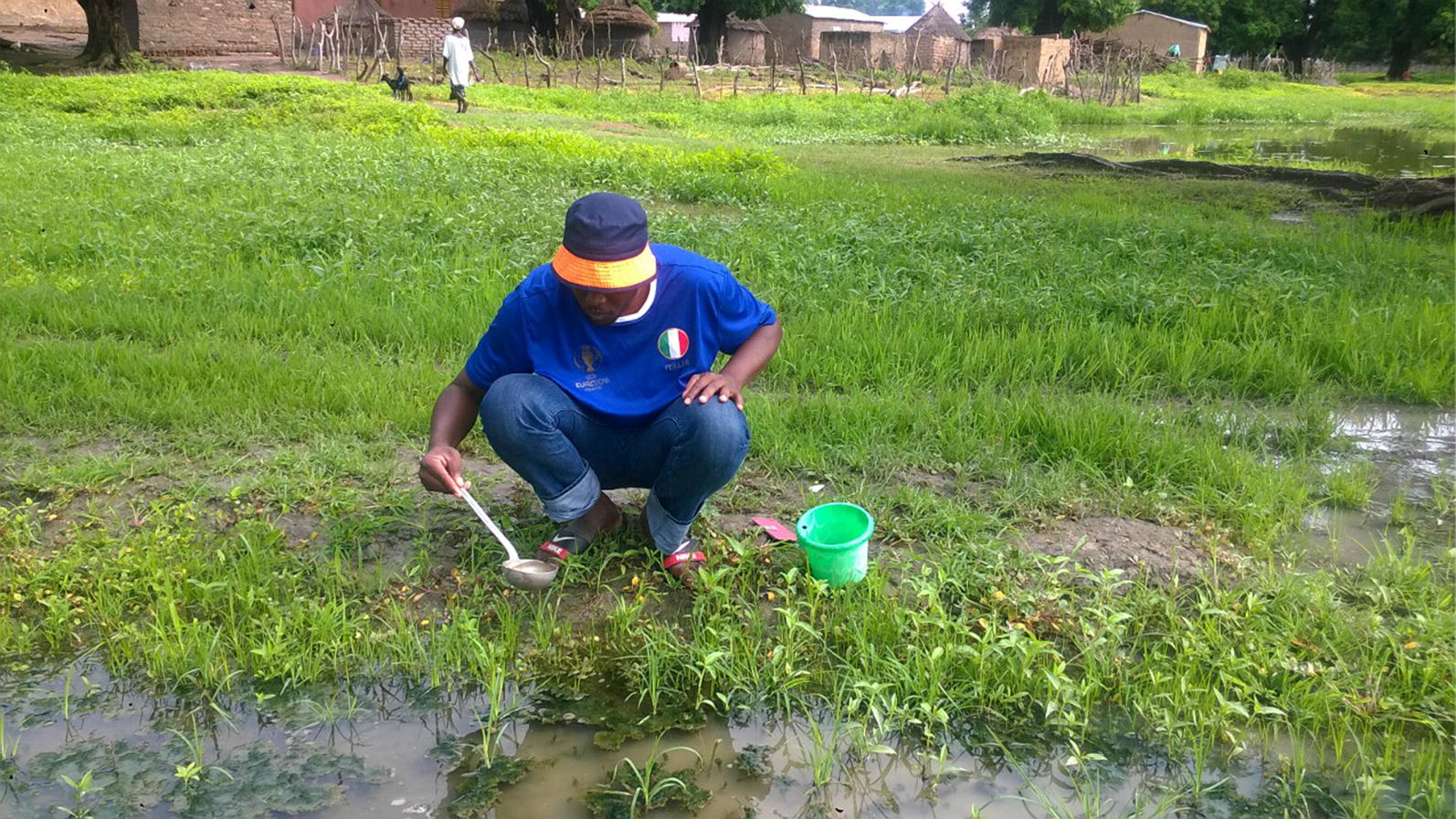

## Background

Malaria vector control relies heavily on the use of long-lasting insecticidal nets (LLINs) and indoor residual spraying (IRS) in Mali. The wide deployment of these control tools is responsible for the current decline in malaria morbidity and mortality globally [1]. For example, LLINs alone are responsible for preventing approximately 68% of malaria deaths in Africa [2]. Four chemical classes of insecticide (organochlorines, pyrethroids, organophosphates, and carbamates) are recommended by the WHO for vector control [3, 4]. Pyrethroids are the only class of insecticide currently used in LLINs and unfortunately, a widespread resistance of the malaria vector is now being recorded with these insecticides. The proportion of malaria-endemic countries that monitored and subsequently reported pyrethroid resistance increased from 71% in 2010 to 81% in 2016 [5]. In addition, resistance to other classes of insecticides (carbamates and organophosphates) has been reported in several West African countries including Mali [6, 7]. Thus, the resistance of malaria vectors to all commonly used classes of insecticide in public health will compromise malaria control efforts in many countries if a good insecticide resistance plan is not put in place [1, 8, 9].

Mali experienced a nationwide distribution of LLINs and the implementation of IRS in selected districts with the support of the US Presidential Malaria Initiative (PMI) and other partners. Different classes of insecticides have been used in the course of this programme. Pyrethroids (lamda-cyhalothrin and deltamethrin) were first used between 2008 and 2010, then replaced with carbamates (bendiocarb) in 2011 and, recently, organophosphates (pirimiphos-methyl) was introduced in this programme. Resistance to carbamates and organophosphates has been well documented in many sub-Saharan countries [10–14]. It was first reported in *Culex quinquefasciatus* from Côte d’Ivoire [15]. Reduced susceptibility to organophosphates (Ops) and carbamates (CMs) was recently observed in *An. gambiae* populations in the centre and the north of Côte d’Ivoire [16] and Benin [17]. The main resistance mechanisms to CMs and OPs documented thus far are either metabolic or target-site mutations. Both resistance mechanisms are widespread in *Anopheles gambiae* (*sensu lato*) and *Anopheles funestus*, the main malaria vectors in Africa. Metabolic resistance to carbamates is often conferred by the upregulation of detoxification genes such as cytochrome P450s [18, 19]. Target-site resistance to CMs and OPs is conferred by a single point mutation causing acetylcholinesterase inhibition [20, 21]. The mutation encoded by the *ace*-*1*^*R*^ gene induces a substitution from a glycine (GGC) codon to a serine (AGC) codon at position 119 (mutation G119S) in resistant mosquitoes [21]. An alternative mutation of the *ace-1*^*R*^ gene (F290V) has been described in *Culex pipiens*; the latter is due to a substitution of phenylalanine (F) by valine (V) at position 290 [22].

In Mali, scarce data exist on carbamate and organophosphate resistance and the different mechanisms underlying this resistance. This study reports the presence of bendiocarb resistance in wild populations of *An. arabiensis*, *An. coluzzii* and *An. gambiae* (*s.s.*) in Mali and further investigates the allelic frequency of the G119S mutations in these wild populations of *An. gambiae* (*s.l.*).

## Methods

### Study area

In Mali, the vector control strategy is based on LLINs, which was scaled up to universal coverage in 2014. In addition, from 2008 to 2016, Koulikoro district was one of the selected districts for IRS implementation in conjunction with LLINs. In the course of this IRS campaigns, three classes of insecticide (pyrethroids, carbamates and organophosphates) were used.

**Fig. 1:**
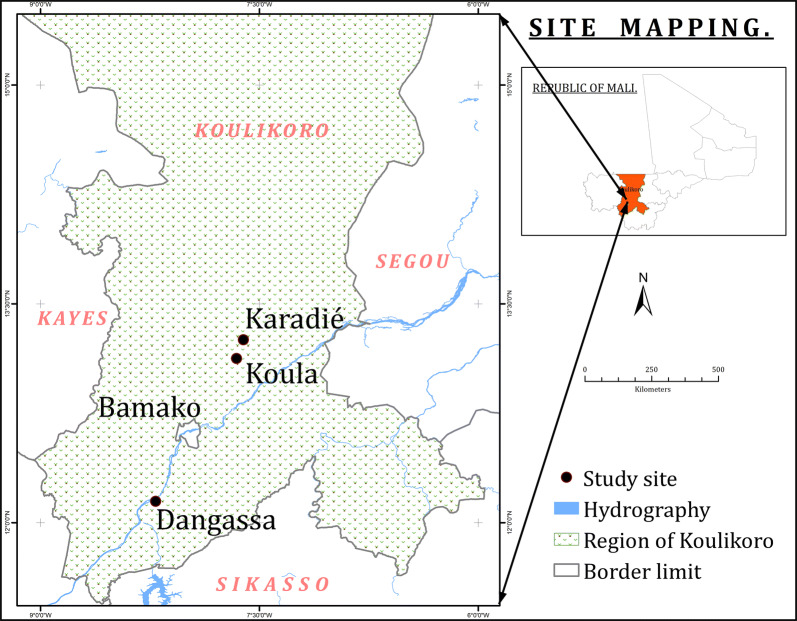
Map showing the study sites in administrative region of Koulikoro, Mali

This study was conducted in the village of Dangassa (8° 12′ 38″ W, 12°8′ 46″ N) located in the health district of Ouéléssébougou, along the River Niger in the Sudan Savanna region, and the villages of Koula (7° 39′ 22″ W, 13° 7′ 28″ N) and Karadie (7° 36’34″ W, 13° 16′ 12″ N) in the health district of Koulikoro, in the Sudano-Sahelian region (Fig. 1). Malaria transmission mainly occurred during the rainy season (June to October) in both areas; however, the presence of the River Niger in the district of Ouéléssébougou makes it a year-round transmission area.

The main economic activity in the each of the three localities is agriculture. The main crops are cotton, millet, peanuts and soybeans. Multiple classes of pesticides such as pyrethroids, carbamates and organophosphates are used to protect these crops against agricultural pests. *Plasmodium falciparum* is the predominant malaria parasite species found in all of the localities and is transmitted by species of the *An. gambiae* species complex. The prevalence of malaria infection varies from 40% to 50% in children under 5 years-old. Resistance to pyrethroids has been reported in all the three villages.

### Mosquito collections

Larvae (L1 to L4) of *An. gambiae* (*s.l.*) were collected in different types of breeding habitats including puddles, brick pits, ponds, tires and animal footprints found in and around each village using the “dipping” technique as described by Service [23]. Collected larvae were transported to the insectary of ICER-Mali in Bamako, where they were maintained at 28 ± 2 °C and a relative humidity of 72 ± 5%. Larvae were separated into different instars in order to have adult mosquitoes emerging at the same time. Larvae were fed with Tetramin powder (Tetra, Herrenteich, Germany), a fish-food, until pupation stage and transferred into cages for adult production. Cotton wool pads soaked with 10% sugar solution were used for feeding emerging adult mosquitoes.

### Insecticide susceptibility bioassays

Three to five days after emergence, adult mosquitoes were sexed before being subjected to the insecticide susceptibility test following the standard protocol of the World Health Organization [24, 25]. All tests were performed in the morning between 8:00 h and 12:00 h in a controlled air-conditioned room at 27 ± 1 °C and a relative humidity of 80%. Test papers impregnated with recommended diagnostic concentrations of 0.1% bendiocarb were used. Insecticide papers were obtained from the WHO reference centre at the Vector Control Research Unit, University Sains Malaysia. Mosquitoes were transferred from holding tubes to test tubes laminated with the insecticide-impregnated paper for one hour. After one-hour of exposure to the insecticide, mosquitoes were transferred again into holding tubes (no insecticide), kept in the insectary and fed with sugar solution. Mortalities were recorded at 24 h post-exposure to insecticide.

Alive mosquitoes were kept in 1.5 ml Eppendorf tubes containing RNA-later (Sigma-Aldrich, Steinheim, Germany) and the dead mosquitoes were kept in tubes with silica gel. All preserved mosquitoes were stored in a freezer at -20 °C for molecular identification and resistant gene screening. Data from molecular identification were used for subgrouping dead and alive mosquitoes into species of the *An. gambiae* complex, and determining susceptibility profiles of each species of this complex, i.e. *An. arabiensis*, *An. coluzzii* and *An. gambiae* (*s.s.*) to bendiocarb in each surveyed village.

### Molecular identification of *An. gambiae* (*s.l.*)

For each village, dead and alive subgroups of *An. gambiae* (*s.l*.) exposed to bendiocarb were molecularly identified and separately sorted to record susceptibility profiles to bendiocarb of wild *An. arabiensis*, *An. coluzzii* and *An. gambiae* (*s.s.*). For this experiment, the genomic DNA of both alive and dead mosquitoes was extracted using the DNA extraction protocol described by Livak [26]. Specific DNA sequences were amplified using the technique of Santolamazza et al. [27] for identification of *An. arabiensis*, *An. coluzzii* and *An. gambiae* (*s.s.*).

### Screening of *ace-1* mutation in *An. gambiae* (*s.l.*) from the study sites

We determined the allelic frequency of *ace-1* in each member of the *An. gambiae* complex, i.e. *An. arabiensis*, *An. coluzzii* and *An. gambiae* (*s.s.*) using the TaqMan SNP genotyping assay. The entire target marker *ace-1*^*R*^ (G119S) was diluted in a total volume of 10 μl containing 2× qPCR Sensimix (Bioline, Cincinnati, USA), 80× primer/probe mix [Ace 1 Forward (5’-GGC CGT CAT GCT GTG GAT-3’); Ace1 Reverse (5’-GCG GTG CCG GAG TAG A-3’); ACE1-VIC (5′-TTC GGC GGC GGC T-3′); ACE1-6-FAM (5′-TTC GGC GGC AGC T-3′)], nuclease-free water and 1 μl template DNA. Probes were labeled with two specific fluorescent dyes, FAM and HEX. The reporter dye (FAM) was used to detect homozygous resistant genotypes (RR), while the quencher fluorescent dye (HEX) was used for the detection of homozygous susceptible genotypes (SS). Both FAM and HEX are specific for the detection of heterozygous resistant/susceptible genotypes (RS). Amplifications were performed in an Agilent MX3000 real-time qPCR machine (Agilent Technologies, Santa Clara, CA, USA) with cycling conditions of 95 °C for 10 min, followed by 40 cycles at 95 °C for 10 s and 60 °C for 45 s. FAM and HEX fluorescence was captured at the end of each cycle and genotypes were called from endpoint fluorescence using the MxPro software (Agilent).

### Linking observed bendiocarb resistance to *ace-1* mutation

To further analyze phenotypic and genotypic associations in observed resistance profiles, we conducted separately, on dead and alive mosquitoes, TaqMan SNP genotyping assays as described above [28]. We determined the allelic frequency of *ace-1*^*R*^ (G119S) in dead and alive mosquitoes post-exposure to bendiocarb. These analyses were conducted at the *Anopheles gambiae* (*s.l.*) level but also disaggregated for each member (*An. arabiensis*, *An. coluzzii* and *An. gambiae* (*s.s.*)).

### Data analysis

Following exposure of mosquito populations to bendiocarb, those with recorded mortalities between 98–100% were rated as susceptible. Populations showing mortality below 98% were rated as suspicious of resistance and populations showing mortality below 90% were rated as resistant [25]. Genotype distributions were recorded in an Excel datasheet (Microsoft Office 2017; Microsoft Corporation, Redmond, USA) and analysis performed using SPSS 25.0. Allelic frequencies were calculated using the following formula ƒ(R) = (2n.RR + n.RS)/2N, where n is the number of mosquitoes of a given genotype, RR represents the homozygote resistance allele, RS represents the heterozygote resistance allele, SS represents the susceptible allele, and N is the total number of mosquitoes tested. After PCR identification for each species per study site, the cumulative binomial exact test was used to test the significance of the resistance on the sample size where the recommended number was not reached. The Chi-square test or Fisher’s exact test where appropriate were used to test for significant associations between *ace-1* genotypes and observed phenotypic resistance for the various exposed populations of *An. gambiae* (*s.l.*). The logistic regression model, with the alive status as the dependent variable, was used to measure the adjusted association between the independent variables (species and study site) and the dependent variable, and to compare different categories within each independent variable.

## Results

### Molecular identification within the *An. gambiae* complex in the three surveyed localities

**Fig. 2:**
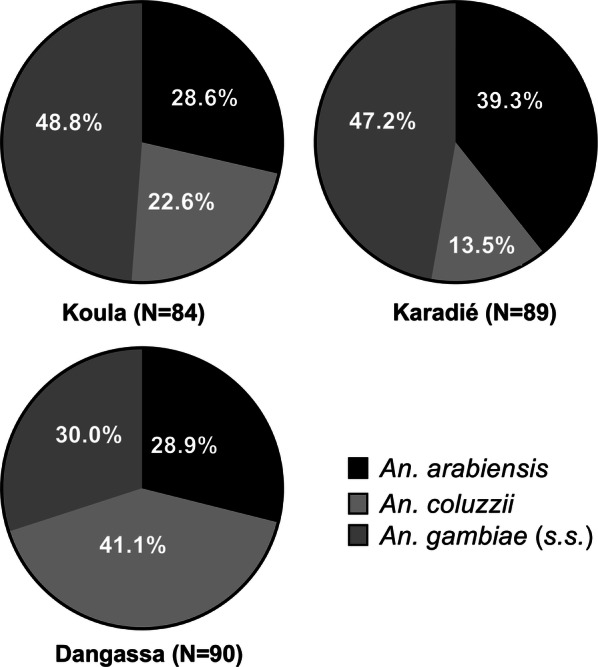
Frequency distribution of species of the *Anopheles gambiae* complex in wild populations exposed to diagnostic doses bendiocarb in all sites in 2016

Out of the 263 specimens of *An. gambiae* (*s.l.*) analyzed by PCR in the three investigated villages 110 (41.98%) were identified as *An. gambiae* (*s.s.*), 85 (32.3%) as *An. arabiensis* and 68 (25.9%) as *An. coluzzii.* Species composition of *An. gambiae* (*s.l.*) varied significantly between study sites (*χ*^2^ = 20.36, *df* = 4, *P* < 0.0001). *Anopheles gambiae* (*s.s.*) was predominant in Koula (48.8%, 41/84) and Karadié (47.2%, 42/89) (Fig. 2). *Anopheles coluzzii* was the most prevalent species in Dangassa (41.1%, 37/90) followed by Koula (22.6%, 19/84), while *An. arabiensis* was prevalent in Karadié (39.3%, 35/89) followed by Dangassa (28.9%, 26/90). In all other sites, *An. gambiae* (*s.s.*) was predominant. The three species were found in sympatry in all sites.

### Susceptibility to carbamates

**Fig. 3:**
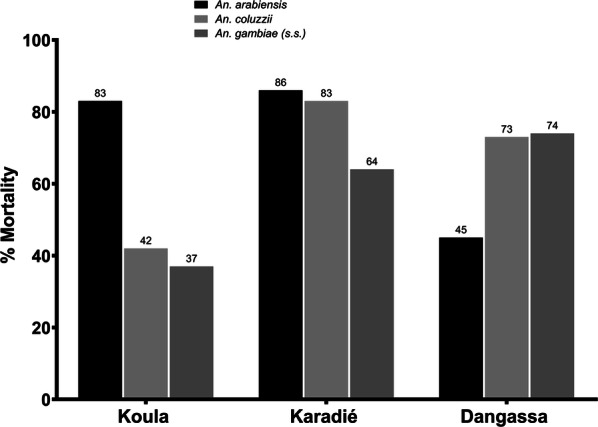
Mortality of wild populations of species of the *Anopheles gambiae* complex exposed to diagnostic doses of bendiocarb in all sites in 2016

Resistance of *An. gambiae* (*s.l.*) to bendiocarb was recorded in the three surveyed localities (Table 1, Fig. 3). In bivariate analysis, the rating of the populations as resistant was significant (*P* < 0.05) for all species with respect to localities within all strata (by locality and by species), except for *An. arabiensis* in Koula and Karadié (*P* = 0.21 and *P* = 0.27, respectively; testing the hypothesis of mortality < 90%) and *An. coluzii* in Karadié (*P* = 0.34), where the rating of the populations as suspicious resistant was significant (*P* < 0.05; testing the hypothesis of mortality < 97%). The highest mortality rate was observed within the latter three strata (83.33%, 85.71% and 83.33%, respectively, see Table 1). The resistance levels of *An. gambiae* (*s.s.*) were significantly high (*P* < 0.05) in all three localities. When this resistance was analyzed with respect to species of the *An. gambiae* complex, we recorded some variations in resistance patterns within the three species in the three localities (Fig. 3). All species populations were significantly rated as resistant in Dangassa (all *P* < 0.05). Highest mortalities were recorded for *An. arabiensis* in the localities of Koula and Karadié (83% and 86%, respectively).

**Table 1:** Binomial exact test for significant rated resistant and suspicious resistant in small sample size situations

Resistance according % dead	Locality	*An. arabiensis*	*An. coluzzii*	*An. gambiae* (*s.s*.)
		% (*P*-value)	% (*P*-value)	% (*P*-value)
Ho: *P*o = 0.9; Ha: *P* < 0.9 (rated resistant)	Koula	83.33 (0.2124)^a^	42.10, (< 0.0001)	36.59 (< 0.0001)
	Karadié	85.71 (0.2693)^a^	83.33 (0.3410)^a^	64.27 (< 0.0001)
	Dangassa	53.85 (< 0.0001)	72.97 (0.0027)	74.07 (0.0147)
Ho: *P*o = 0.97; Ha: *P* < 0.97 (rated suspicious)	Koula	83.33 (0.0053)		
	Karadié	85.71 (0.0037)	83.33 (0.0486)	
	Dangassa			

^a^Not significant at 0.05 level for rated resistant but significant for rated suspicious resistant

The logistic regression models testing adjusted association between bendiocarb resistance (low mortality rate) with the species and with the localities were globally both significant (*P* = 0.036 and *P* = 0.006, respectively). There was a significant difference between the mortality rate of *An. arabiensis* compared to *An. gambiae* (*s.s.*) (OR = 0.439, *P* = 0.011). However, the adjusted difference of the mortality rate between localities were not significant (all *P* > 0.05) (Table 2).

**Table 2:** Association between the mortality and the factors (species and locality), using a logistic regression model

Parameter	*χ*^2^	*df*	OR	95% CI	*P*-value
All species	6.65	2			0.036
*An. arabiensis*	6.46	1	0.439	0.233–0.828	0.011
*An. coluzzii*	1.8	1	0.636	0.328–1.233	0.180
All localities	10.28	2			0.006
Koula	3.79	1	1.867	0.991–3.519	0.053
Karadié	1.51	1	0.651	0.328–1.292	0.220
Constant	1.32	1	0.714		0.250

*Abbreviations*: *χ*^*2*^, Chi-square test; *df*, degrees of freedom; OR, odds ratio; CI, confidence interval

### Distribution of *ace-1* mutation in wild populations of sampled *Anopheles* species

**Fig. 4:**
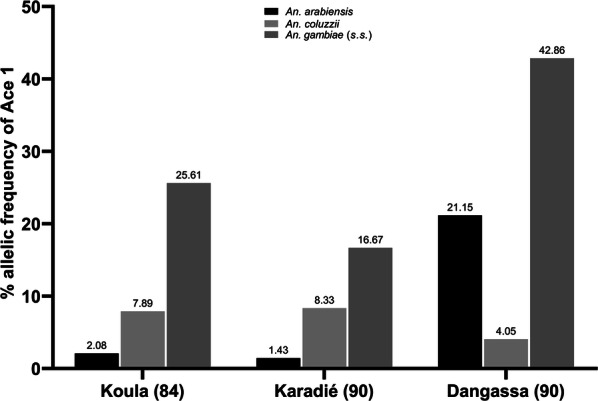
Allelic frequency of the *ace-1* mutation in wild populations of species of the *Anopheles gambiae* complex from the surveyed localities

Two hundred and sixty mosquitoes from Koula (*n*  =  84), Karadié (*n * = 89 ) and Dangassa (*n*  =  90) were assayed for molecular form and analyzed for their *ace-1* genotype (Table 3). All specimens carrying the gene were heterozygotes. Generated data for the allelic frequency of the *ace-1*^*R*^ mutation revealed relatively high frequency of this mutation in wild populations of *An. gambiae* (*s.l.*). from Dangassa compared to Koula and Karadié. At Dangassa, the mutation was dominant in *An. gambiae* (*s.s.*) (42.9%) followed by *An. arabiensis* (21.2%) and *An. coluzzii* (4.1%) with significant differences between species (*χ*^2^ = 10.3, *df* = 2, *P* = 0.006). At Koula, the mutation remained with high frequency in *An. gambiae* (*s.s.*) (25.6%) followed by *An. coluzzii* and *An. arabiensis* (*χ*^2^ = 18.33, *df* = 2, *P* < 0.0001); a similar trend was recorded in Karadié (*χ*^2^ = 11.53, *df* = 2, *P* = 0.003) (Fig. 4). We observed a relatively high frequency of *ace-1* mutation in *An*. *arabiensis* from Dangassa (21.2%) compared to *An. arabiensis* from Koula and Karadié, which had low levels of mutation (2.1% and 1.4%, respectively) (*χ*^2^ = 21.12, *df* = 2, *P* < 0.0001; Fig. 4). For the *An. coluzzii* the difference between the three localities was not significant (*χ*^2^ = 1.05, *df* = 2, *P* = 0.59). In contrast, the distribution of *ace-1* in *An. gambiae* (*s.s.*) was significantly different across the localities with the high presence in Dangassa (42.86%) (*χ*^2^ = 6.31, *df* = 2, *P* = 0.043).

**Table 3:** Acetylcholinesterase genotypes and frequency of *ace-1*^*R*^ mutation of *Anopheles gambiae* (*s.l.*) in 2016

Genotype	Koula (bendiocarb)		Karadié (bendiocarb)		Dangassa (bendiocarb)	
	Alive	Dead	Alive	Dead	Alive	Dead
RR (*n*)	0	0	0	0	0	0
RS (*n*)	25	0	17	0	20	0
SS (*n*)	16	43	5	67	9	61
Exposed individuals (*n*)	41	43	22	67	29	61
*f(R-ace-1*) (%)	30.48	0	38.63	0	31.25	0

*Abbreviations*: RR, homozygote resistance allele; RS, heterozygote resistance allele; SS, susceptible allele

### Association between *ace-1* mutation frequencies and recorded bendiocarb resistance profiles in the three surveyed localities

The analysis of the presence of the resistant allele of *ace-1* in alive and dead individuals following exposure to bendiocarb showed consistent associations between mosquito survivors after bendiocarb exposure and the presence of the mutation (Table 4); indeed, none of the dead mosquitoes carried the mutation. The mutation was never found in the population of individuals dying post-exposure to bendiocarb. The survival of *Anopheles gambiae* (*s.l.*). populations from the three surveyed localities was associated with the presence of the mutation. However, the fact that some survivors did not carry the mutation highlights the presence of other bendiocarb resistance mechanisms in these localities.

**Table 4:** Allelic frequencies of G119S genotypes sorted in wild *An. gambiae* (*s.l*.) populations alive and dead individuals post-bendiocarb exposure in 2016

Locality	*An. arabiensis*		*An. coluzzii*		*An. gambiae* (*s.s*.)	
	% G119S (*n*)		% G119S (*n*)		% G119S (*n*)	
	Alive	Dead	Alive	Dead	Alive	Dead
Koula	12.50 (4)	0 (20)	13.64 (11)	0 (8)	40.38 (26)	0 (15)
Karadié	10.00 (5)	0 (30)	50.00 (2)	0 (10)	46.67 (15)	0 (27)
Dangassa	45.83 (12)	0 (14)	15.00 (10)	0 (27)	42.86 (7)	0 (20)

### Association between *ace-1* mutation frequencies and recorded bendiocarb resistance profiles in members of the *An. gambiae* complex in the three surveyed localities

When data of allelic frequencies of G119S mutation were disaggregated at the species level (Table 4), we recorded an association of survival after bendiocarb exposition and the presence of *ace-1* mutations in *An. gambiae* (*s.s.*). Allelic frequencies of 40.4%, 46.7% and 42.9% were recorded in surviving *An. gambiae* (*s.s.*) populations at Koula, Karadié and Dangassa, respectively. Similarly, most bendiocarb surviving *An. arabiensis* and *An. coluzzii* also carried the G119S mutation, but not at high levels as in *An. gambiae* (*s.s.*) with a few exceptions (Table 4).

## Discussion

Currently, there is growing concern that the widespread resistance of the malaria vector to pyrethroid insecticides reduces the effectiveness of LLINs and IRS and compromises the current decline in malaria morbidity and mortality globally. An insecticide resistance management strategy may help to moderate these effects. The first step of insecticide resistance management is the determination and monitoring of the resistance mechanisms prevailing in the area. In Mali, the target site mutation, specifically the knockdown resistance (*kdr*) allele has been well documented [7, 29, 30] but data on the other resistance mechanisms are scarce. In this study, we assessed the status of *An. gambiae* (*s.l.*) to CMs and determined the frequency of the *ace-1* mutation in the health districts of Koulikoro (with LLINs plus IRS), and Ouéléssébougou (with only LLINs).

### Molecular identification within the *An. gambiae* complex in the three surveyed localities

Our results revealed the sympatric presence of *An. gambiae* (*s.s.*), *An. arabiensis* and *An. coluzzii* in the three sites with different proportions as reported by a number of studies in Mali [7, 31, 32] . *Anopheles gambiae* (*s.s.*) was the major species in Koula and Karadié and *An. coluzzii* in Dangassa. This is certainly related to the types of breeding habitats found in each of the two areas. In Koula and Karadié, larval habitats are rainfall dependent and sunny, such as brick pits, tire prints and puddles, which are preferred by *An. gambiae* (*s.s.*). In Dangassa, the flooded plain that separates the village from the River Niger constitutes the favorable environment for the development of *An. coluzzii* [33, 34].

### Susceptibility to carbamates

All three species of the *An. gambiae* complex (*An. gambiae* (*s.s.*), *An. arabiensis* and *An. coluzzii*) found in the study sites were resistant to bendiocarb in Dangassa, while *An. gambiae* (*s.s.*) was rated as resistant in all the three localities at the significance lavel of *P* < 0.05. There was variation in the level of resistance by species and by area. In Dangassa, cotton and vegetable cropping is very common and therefore the use of agricultural pesticides in addition to the wide deployment of the LLINs may have triggered a selection pressure on mosquitoes, as reported in many studies across West Africa [35–39]. The resistance observed in Koula and Karadié can be attributed to the use of bendiocarb in the IRS campaigns from 2011 to 2013 in these localities. Indeed, previous data from sentinel sites of the National Malaria Control Programme across the country showed low resistance of species of the *An. gambiae* complex to bendiocarb [6, 7]; resistance of species of the complex to the carbamate class of insecticide has been reported elsewhere in Africa in many studies [10, 13, 40, 41].

### Distribution of *ace-1*^*R*^ mutation in wild populations of sampled *Anopheles* species

Our study showed the presence of the G119S mutation in the three species and at all study sites. The allelic frequencies of the G119S mutation were higher in *An. gambiae* (*s.s.*) than in *An. coluzzii* in all study sites. Similar observations were reported in Burkina Faso [42] and in Ghana [12]. However, higher allelic frequencies of the G119S mutation was reported in *An. coluzzii* compared to *An. gambiae* (*s.s.*) in Côte d’Ivoire [11]. In Mali, the only study on the G119S mutation was carried out by Cissé et al. [7] who reported a low frequency of the mutation in these two species. We also noticed a higher frequency of the mutation G116S in *An. arabiensis* in Dangassa, in the Sudan Savanna region, compared to Karadié and Koula, in the Sahel region. Similar observations were made in Burkina Faso [43].

### Association between *ace-1*^*R*^ mutation frequency and recorded bendiocarb resistance profiles in the three surveyed localities

Our study showed that all resistant specimens carried the resistant allele (R) in its heterozygous form. The absence of a homozygous specimen is probably due to the high mortality of resistant homozygotes caused by the G119S mutation. Indeed, many studies [21, 44, 45] reported that homozygous resistant individuals are most likely to die during pupation than susceptible individuals. Therefore, in the area where the *ace-1* resistant allele is present, the resistant mosquitoes will mainly be of the heterozygous (*ace-1* RS) status. Djogbénou et al. [46] reported that the presence of the *ace-1* RS affects the body size of adult mosquitoes, with resistant individuals being smaller than the susceptible ones. Our results showed that the G119S mutation was present in alive specimens of all study sites. This observation is in accordance with other studies [47, 48] supporting the association of the G119S mutation with the survival of *An. gambiae* (*s.l.*) populations. Our results also showed that the *ace-1* gene frequency was associated with *An. arabiensis* resistance to bendiocarb in Dangassa, *An. coluzzii* in Koula and Dangassa, and *An. gambiae* (*s.s.*) in all surveyed localities.

## Conclusions

This study documented the resistance levels to bendiocarb of all three species of the *An. gambiae* complex in Mali. We have also shown the link between the molecular mechanism and the observed phenotypic resistance and implicated the G119S mutation in bendiocarb resistance in *An. gambiae* (*s.l.*) populations. Periodic updates of data on resistance of major vectors are required for a rational planning of insecticide deployment and insecticide resistance mitigation.

## Data Availability

Data supporting the conclusions of this article are included within the article. The datasets analyzed during the present study are available from the corresponding author upon reasonable request.

## Abbreviations

NMCP: National Malaria Control Programmes
WHO: World Health Organization
LLINs: long-lasting insecticidal nets
IRS: indoor residual spraying
*ace-1*^*R*^: acetylcholinesterase-1 resistance
SNP: single-nucleotide polymorphism
R: resistant
S: susceptible
